# The Thrombopoietin Receptor: Structural Basis of Traffic and Activation by Ligand, Mutations, Agonists, and Mutated Calreticulin

**DOI:** 10.3389/fendo.2017.00059

**Published:** 2017-03-31

**Authors:** Leila N. Varghese, Jean-Philippe Defour, Christian Pecquet, Stefan N. Constantinescu

**Affiliations:** ^1^Ludwig Institute for Cancer Research, Brussels Branch, Brussels, Belgium; ^2^SIGN Pole, de Duve Institute, Université catholique de Louvain, Brussels, Belgium; ^3^Department of Clinical Biology, Cliniques universitaires St Luc, Université catholique de Louvain, Brussels, Belgium

**Keywords:** thrombopoietin, thrombopoietin receptor, JAK2, calreticulin, N-glycosylation, myeloproliferative neoplasms, megakaryocytes, congenital amegakaryocytic thrombocytopenia

## Abstract

A well-functioning hematopoietic system requires a certain robustness and flexibility to maintain appropriate quantities of functional mature blood cells, such as red blood cells and platelets. This review focuses on the cytokine receptor that plays a significant role in thrombopoiesis: the receptor for thrombopoietin (TPO-R; also known as MPL). Here, we survey the work to date to understand how this receptor functions at a molecular level throughout its lifecycle, from traffic to the cell surface, dimerization and binding cognate cytokine *via* its extracellular domain, through to its subsequent activation of associated Janus kinases and initiation of downstream signaling pathways, as well as the regulation of these processes. Atomic level resolution structures of TPO-R have remained elusive. The identification of disease-causing mutations in the receptor has, however, offered some insight into structure and function relationships, as has artificial means of receptor activation, through TPO mimetics, transmembrane-targeting receptor agonists, and engineering in dimerization domains. More recently, a novel activation mechanism was identified whereby mutated forms of calreticulin form complexes with TPO-R *via* its extracellular N-glycosylated domain. Such complexes traffic pathologically in the cell and persistently activate JAK2, downstream signal transducers and activators of transcription (STATs), and other pathways. This pathologic TPO-R activation is associated with a large fraction of human myeloproliferative neoplasms.

## Introduction

The cytokine thrombopoietin (TPO) is the chief regulator of megakaryocyte (MK) and platelet production, signaling *via* its receptor, MPL (TPO-R). The receptor was first identified in 1992 (1) and its ligand, TPO, was cloned not long after by several independent groups (2–7). Since then much effort has been made to characterize TPO-R and understand its function in normal and pathological hematopoiesis.

Thrombopoietin acts by binding to the extracellular portion of partially predimerized cell surface TPO-R, thought to cause a change in the receptor dimer arrangement and/or monomer–dimer equilibrium, which initiates a cascade of signaling events within the target cell. TPO-R has no intrinsic kinase activity and utilizes the Janus kinase (JAK) family of proteins to transduce signal from the extracellular cytokine to the nucleus within the cell. Two JAKs, JAK2 and TYK2, constitutively associate with the cytoplasmic tail of TPO-R and are phosphorylated upon TPO signaling (8–10), although TYK2 does not seem to be essential in this pathway (11, 12). These activated JAKs then phosphorylate the receptor itself, as well as signal transducer and activator of transcription (STAT) 1, 3, and 5 (13–15), and additionally can activate the mitogen-activated protein kinase (MAPK) (16) and phosphatidylinositol-3 kinase (PI3K) pathways (17).

TPO-R is expressed predominantly on the surface of MKs, platelets, hemangioblasts, and hematopoietic stem cells (HSCs) (18, 19). This pattern of expression is indicative of the two key functions of TPO: the regulation of platelet production (20–22) and the maintenance of HSCs (20, 23, 24). These important and non-redundant effects of TPO can be seen in mice where either *Tpo* or *Mpl* have been genetically deleted. A dramatic reduction in platelet and MK numbers—to about 10% of that of wild-type mice—can be observed in these mice, while mature blood cells are not affected in other lineages. *Mpl*^−/−^ mice additionally have an increase in circulating Tpo (20, 22), but otherwise all these mice are healthy and viable. In humans, however, a lack of functional MPL results in congenital amegakaryocytic thrombocytopenia (CAMT), a rare condition in which infants develop a platelet and MK deficiency that, untreated, leads to multilineage failure (25–27). This difference between the two species in fundamental requirement for MPL for sustainable hematopoiesis suggests that perhaps compensatory mechanisms exist in mice, which are not present in humans. Some reduction in myeloid cells in *Mpl*^−/−^ mice as they age (23), however, does bring up the possibility that the lifespan of mice is too short to observe a complete exhaustion of HSCs.

While a complete loss of TPO-R leads to thrombocytopenia, other mutations in *MPL* have been identified in patients with increased platelet numbers, in disorders such as familial thrombocytosis and two of the myeloproliferative neoplasms (MPNs), essential thrombocythemia (ET), and myelofibrosis (MF) (28–35). Understanding physiological functions of TPO-R and how this is altered in disease is therefore clearly desirable to develop effective therapeutics to treat such conditions.

## Domain Structure

**Figure 1:**
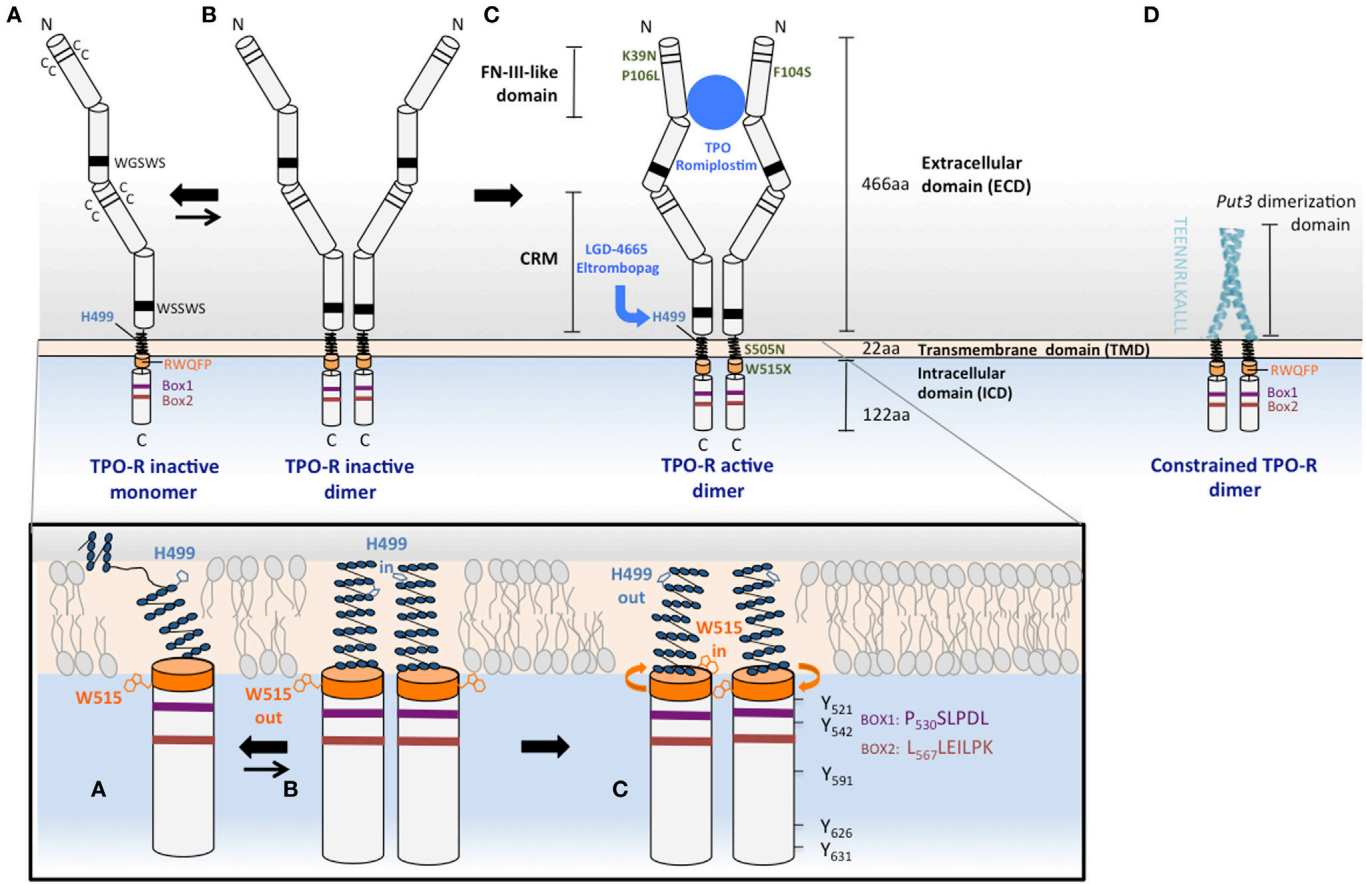
**Mechanisms of TPO-R activation**. Upper panel: **(A)** the human TPO-R has common cytokine receptor features, such as pairs of cysteines and conserved motifs in the extracellular domain (ECD), and also contains unique properties that prevent its self-activation and make it exist as a monomer or monomer–dimer equilibrium. The membrane distal ECD, the H499 in the transmembrane domain (TMD), and the RW_515_QFP motif (orange) play important roles in maintaining the receptor inactive in the absence of ligand. H499 creates a break in the helical extracellular juxtamembrane and the TMD, regulating dimerization and preventing oncogenic activation by mutants that are found active in other species. W515 anchors the TMD in the membrane, tilts the TMD relative to the bilayer structure, and is oriented outside the dimer interface in the inactive orientation, W515 “out,” H499 “in”; activation will result in the opposite orientation: W515 “in,” H499 “out.” **(B)** The inactive dimeric state of the TPO-R (less energetically favored compared to monomer) exhibits helical structure that is restored above H499 and adopts the H499 “in” and W515 “out” with respect to the interface of more parallel TMD dimers. **(C)** TPO, TPO analogs such as romiplostim, small molecule TPO agonists, such as eltrombopag, and some activating mutations such as W515L or S505N found in MPNs are able to shift the equilibrium from this unique inactive dimer toward active dimers, where W_515_ is rotated more in the interface (“in”) and H499 is maintained more outside (“out”). TPO-R active dimers lead to positioning of JAK2 proteins in a conformation conducive for activation, transphosphorylation, and subsequent phosphorylation of tyrosine residues that are located in the cytoplasmic tail of the receptor and in JAK2 itself. The TPO-R F104S mutation results in receptor that is unable to bind TPO, therefore, this mutant receptor cannot be activated by TPO. Other mutations such as K39N or P106L produce a low level of TPO-R cell surface localization that is sufficient for signaling, but that does not efficiently clear TPO from circulation. **(D)** Replacement of the ECD and fusion of an artificial dimeric structure (coiled-coil domain of the Put3 transcription factor) to progressively shorter TMDs impose individually all seven possible dimeric orientations of the TPO-R TMD. The sequence depicted vertically is the C-terminus of the Put3 coiled-coil domain that is fused to different residues of the TMD to create dimers adopting different interfaces and probe function of each such interface (36). Lower panel: cartoons representing the helical TMD monomeric conformation depicted in **(A)** and the helical TM dimer orientations in **(B)** (His “in”) and in **(C)** (His “out”). Conserved Box1, Box2, and tyrosine residues are shown for the cytosolic domain of TPO-R in **(C)**.

TPO-R is 635 amino acids in length and has three functional domains: an extracellular portion involved in cytokine binding, a transmembrane domain (TMD), and a cytoplasmic domain, likely largely unstructured, which binds JAKs and other signaling molecules, such as STATs (Figure 1C, upper panel). Membrane proteins are notoriously difficult to crystallize, and TPO-R is no exception. Consequently, much of our idea of TPO-R’s structure is based on studies of related cytokine receptors for which some parts of their structures are known, such as erythropoietin receptor (EPO-R) and growth hormone receptor (GH-R).

## ECD: The Sensor

The extracellular domain (ECD) of the receptor is thought to follow a similar arrangement as the EPO-R and related receptors, except that it is composed of two adjacent pairs of fibronectin-III (FNIII)-like domains rather than the single pair of EPO-R (Figure 1). Each FNIII-like domain consists of a series of seven antiparallel β-strands that stack in a β-sandwich. By homology with known related structures, the hinge region connecting the FNIII-like domains within a pair [also known as a cytokine receptor module (CRM)] forms the interface for cytokine binding (37). Several studies have indicated that the membrane distal CRM (CRM-1) is essential for interaction with TPO (38), since its deletion (or replacement with the membrane proximal pair) abrogates TPO binding and possibly has a role in preventing signaling in the absence of TPO (39).

Indeed, most of the ECD of murine TPO-R (Tpo-R) is missing in the murine myeloproliferative leukemia virus envelope protein (v-mpl), which, as its name suggests, drives a myeloproliferative leukemia in mice *via* an activated truncated form of TPO-R. This oncoprotein is a fusion of 100 amino acids derived from the Friend murine leukemia virus envelope sequence and a 184 amino acid portion of Tpo-R (residues 440–623), comprising the transmembrane and cytoplasmic regions of the receptor, but lacking N-terminal region of the receptor, including the membrane distal CRM, and most of the membrane proximal CRM (CRM-2) (40). It is unclear, however, whether it is the absence of CRM-1 or the presence of a portion of the Friend murine leukemia virus, or a combination of the two, that brings about dimerization and activation of the truncated Tpo-R.

The ECD mutation F104S, located in the membrane distal FNIII-like domain of CRM-1, is found in CAMT patients, and studies of this mutant receptor in BaF3 cells suggest that an essential ligand-binding site is disrupted, as cells are neither stimulated by TPO nor internalize the cytokine (41), despite the presence of the receptor on the cell surface. The loss of ligand binding on mutagenesis of TPO-R F45, L103, and P257 indicates that these residues as well, predicted to be located within loops that form a hinge region, are likely to contribute to TPO binding (42). However, direct evidence *via* an experimentally derived structure of TPO-R in complex with TPO is necessary to validate this result.

## TMD: The Control Center

The TMD is a helical section of the receptor that anchors it to the cell surface. Receptor dimerization is necessary for receptor activation, and the role of the TMD in activation has been highlighted by the identification of activating mutations in this domain and the adjacent juxtamembrane region, as well as by biochemical studies of the dimerized transmembrane and cytoplasmic domain orientation.

A mutation at the juxtamembrane W515 residue was first identified as activating in cell lines by Staerk and colleagues (43) (W515A mutation) and by Pikman and colleagues (29) in patients with acquired ET and MF (W515L mutation). While mutations of this residue to lysine and leucine are the most prevalent in patients, extensive mutagenesis of this residue has further demonstrated that all residues (except tryptophan, cysteine, and proline) constitutively activate the receptor when placed at this position (44). W515 has a key role in preventing activation in the absence of cytokine binding. This residue is part of the amphipathic helical motif RWQFP, which is directly adjacent to the C-terminal end of the TMD (Figure 1A, lower panel). This motif is unique to TPO-R, and its deletion or mutation activates the receptor in the absence of ligand (43). NMR studies of transmembrane peptides indicate that mutation of W515 decreases the tilt angle relative to the lipid bilayer normal, bringing the dimer into an active conformation (45).

Another clinically relevant TMD mutation, which activates TPO-R is S505N, and is found in patients with hereditary and rare sporadic cases of thrombocythemia. This mutation was first reported in a Japanese family (28) and was later found with some frequency in Italian patients with the same disorder (46, 47). S505N induces stable active receptor dimers (28), and mutational studies show that H499 near the N-terminus of the human TPO-R TM domain is protective against the constitutive activation driven by several other asparagine mutations that fully activate the murine receptor (48). The first NMR studies on the transmembrane helix of TPO-R found that its helical region is not continuous but rather unexpectedly contains several non-helical residues in the sequence preceding H499 in the human form of the receptor (Figure 1A, lower panel), although the same region in the murine receptor is helical (49). Both the S505N mutation and the TPO-R small molecule agonist eltrombopag, which binds to H499 (Figure 1C, upper panel), however, induce helical structure into the region, stronger dimerization, and receptor activation, although eltrombopag is not effective on the murine receptor as it lacks H499 (48).

Studies of forced dimerization at different orientations of the TMD (50, 51) indicate that cytokine-induced dimerization is not simply a binary on/off switch in the case of TPO-R, but rather there are a number of possible “active” outputs.

These studies of intracellular domain orientation of TPO-R, similar to those performed earlier of EPO-R (36), suggest that the geometry of these regions play a key role in deciding both the quality and the quantity of the resulting signal (50, 51). One approach has been to use a coiled-coil dimerization domain that is derived from the yeast transcription factor *Put3* and fused to the intracellular domain of Tpo-R (Figure 1D, upper panel) (51). These fusion products are therefore forced to dimerize constitutively and do not require ligand to activate downstream signaling pathways. Different orientations of the two intracellular domain chains could then be systematically altered with the progressive deletions in the juxtamembrane region, which would be predicted to induce rotation within the receptor dimer. In this way, the seven possible relative orientations of the transmembrane and cytoplasmic domains could be imposed. Unlike EPO-R, where these experiments found essentially only a single symmetrical dimeric orientation that was able to signal, TPO-R had several conformations that supported cell proliferation, with differing signaling output and biological effects, including cell–cell adhesion and MK differentiation.

The concept that rotations of the dimeric receptor TMDs drive TPO-R activation, rather than dimerization alone, is supported by other independent approaches, such as TOXCAT (ToxR-mediated activation of chloramphenicol acetyltransferase reporter assay) and cysteine cross-linking assays (50). From these results, the authors propose three different interfaces in the TPO-R TMD dimer: one active, one inactive, and one intermediate activity.

## Cytoplasmic Domain: The Transmitter

The portion of TPO-R that extends into the cytoplasm is the region of the receptor which binds JAKs, as well as interacting with other intracellular proteins, notably signal transducers, such as STATs. The receptor has five cytoplasmic tyrosine residues that, when phosphorylated, serve as potential docking sites for SH2-domain-containing proteins, including STATs and LNK. Accordingly, several studies have attempted to determine a function for each of these five sites [Y521, Y542, Y591, Y626, and Y631; also referred to as Y7, Y28, Y77 (78 in mouse), Y112, and Y117, numbered with respect to the cytoplasmic domain alone] and identify proteins interacting with these phosphorylated tyrosines.

Y521 and Y591 have been implicated in the negative regulation of TPO signaling (52), the former as part of a lysosomal targeting motif, and the latter *via* an interaction with adaptor protein AP2, which mediates receptor internalization through recruitment of clathrin (53). Aside from this lysosomal degradation, activated TPO-R is also subject to degradation by the proteasome, undergoing ubiquitination *via* the E3 ubiquitin ligase CBL, which targets the receptor on the cytoplasmic residues K553 and K573 (54).

In contrast to most other cytokine receptor superfamily members that exhibit short half-lives, TPO-R is long-lived both at the cell surface and in intracellular stores (55). Its traffic to the cell surface and stability is promoted by JAK2 (55). A consequence of this prolonged half-life is observed in its ability to be recycled (56). Surface biotinylation studies have shown that reappearance of cell surface TPO-R after internalization is due to recycling of the internalized receptor (56) and not transport of stored receptor in intracellular pools (57). This recycling process is blocked in cells that coexpress TPO-R and JAK2 V617F, leading to cell surface TPO-R downmodulation (57).

The membrane distal tyrosines Y626 and Y631 (also denoted cytoplasmic residues Y112 and Y117) function as positive regulatory sites (52). The murine homolog of Y626 is required for cellular differentiation and the phosphorylation of Shc, the adaptor that links receptor activation to the MAPK pathway (58). Y626 additionally is necessary for the constitutive signaling in MPN driven by the mutation W515A (59).

Although the cytoplasmic domains of cytokine receptors show little sequence similarity, they do share two conserved elements, Box1 and Box2 (60). These motifs are involved in binding to JAKs and signal transduction (61, 62). Box1 is essential for JAK binding and activation, and this motif centers on two evenly spaced proline residues (PXXP). Upstream from the Box1 sequence, many cytokine receptors possess conserved hydrophobic residues at positions −1, −2, and −6 (63). These residues form a “switch motif,” which is required for cytokine-induced JAK2 activation but not for JAK2 binding (63, 64). Located downstream from Box1, Box2 is less well defined by an increased serine and glutamic acid content, along with hydrophobic residues, and its presence is not always necessary for a proliferative signal (61, 65, 66).

The cytoplasmic region of TPO-R, just as for other cytokine receptors, is considered to lack in large part secondary structure, especially around tyrosines that are phosphorylated, and this flexibility likely accounts for its imperviousness to crystallization attempts. As a consequence, these motifs have, until recently, only been defined indirectly through mutagenesis studies. However in the last few years, clever strategies first used by the Lupardus group to cocrystallize cytokine receptor cytoplasmic regions with FERM and SH2 domains of JAKs, either as a fusion protein or as distinct entities, have proven to be effective. To date, the structures of a section of the cytoplasmic domain of IFNAR1 (PDB 4PO6) (67), IFNLR1 (PDB 5IXD; 5L04) (68, 69), and an IFNLR1:IL10RA chimera (PDB 5IXI) (68), together with their cognate JAKs (TYK2 for IFNAR1 and JAK1 for IFNLR1 and IL10R) have been determined. These first snapshots allow us to visualize the structural basis of the interaction between cytokine receptors and their JAKs and give context to the box motifs.

It had previously been assumed that the JAK FERM domain alone participated in receptor binding (70–72), although studies of JAK1 and gp130 implicated the SH2 as well (73). The structural data, however, when taken together, show Box1 binds to the FERM domain, while Box2 interestingly binds to the SH2-like domain of TYK2, in a manner reminiscent of a phosphotyrosine peptide to a classical SH2 domain (67). Whether a similar binding mode is used by TPO-R is yet to be seen, as those structures concern type II cytokine receptors, while TPO-R is a type I cytokine receptor. Indeed, one might speculate that variations in binding sites and their respective affinities determine the specificity of particular cytokine receptors for particular JAKs.

How TPO-R interacts with JAK2 is particularly relevant in the context of disease driven by mutations in JAK2, specifically JAK2 V617F, which occurs in about half of patients with ET and MF, and most patients with polycythemia vera (34, 74–77). These three diseases are collectively known as MPNs, and in *in vitro* (78), as well as in a transgenic JAK2 V617F mouse model of MPN (79), the disease phenotype is dependent on the expression of TPO-R. Although the cell surface expression of TPO-R is downregulated in the presence of JAK2 V617F, as discussed above, there is sufficient receptor to allow constitutive activation.

This suggests TPO-R could be a good therapeutic target for the treatment of MPN, either by interfering with its interaction with JAK2 V617F or through suppression of TPO-R expression. In addition to these two approaches, a TPO-R antagonist has also recently been studied as a therapeutic for MF (80), exploiting the requirement of TPO-R expression in HSCs for maintaining a reservoir of HSCs and preventing exhaustion (81).

A detailed understanding of the TPO-R and JAK2 complex and the specificity of this particular cytokine receptor for this particular JAK is likely to be useful in determining a strategy to curb pathological signaling *via* JAK2 V617F while minimizing off-target effects. Modifying TPO-R expression, on the other hand, could be problematic, due to its role in regulating TPO levels.

## TPO-R Expression and TPO Regulation

**Figure 2:**
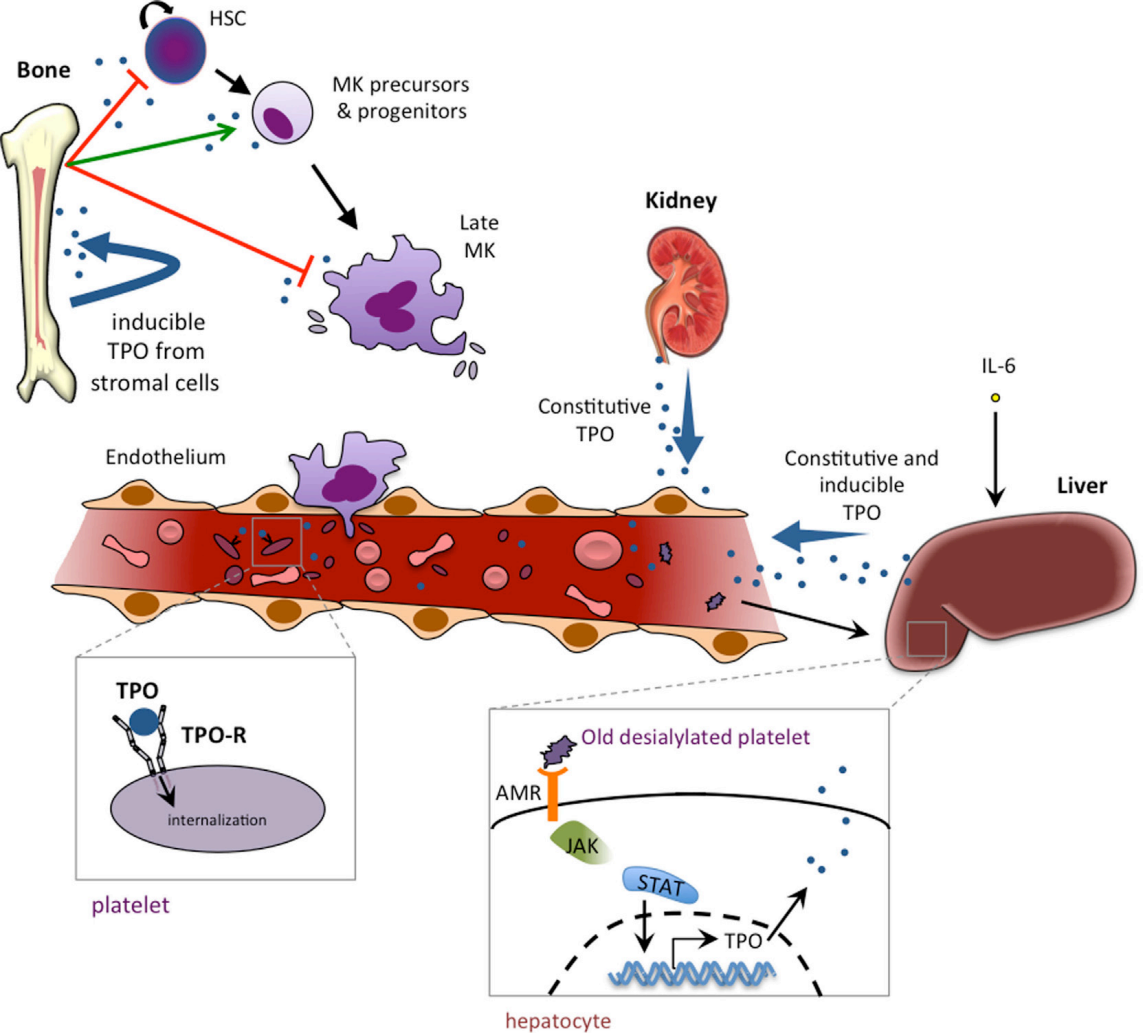
**Thrombopoietin (TPO) production, function, and homeostasis**. TPO is mainly produced by liver parenchymal cells and by endothelial cells of the liver sinusoids. TPO production can also occur in proximal tubule cells of the kidney and in bone marrow stromal cells. The production is mainly constitutive (with no change in TPO mRNA levels), and regulation is mediated by negative feedback of the TPO-R-expressing platelet pool that internalizes and destroys free circulating TPO. IL-6 is able to induce hepatic TPO mRNA production in reactive/inflammatory thrombocythemia. Binding of old desialylated platelets to the hepatic Ashwell–Morell receptor (AMR) has been shown to promote liver TPO mRNA production. TPO plays a dual role in hematopoiesis, with an inhibiting role (red arrow) that occurs at the two extremes of the hematopoietic process, keeping stem cells quiescent and promoting a cell cycle arrest in late megakaryocytes (MKs), and a positive role in stimulating immature precursors (CMP and MEP) and promoting growth of very early progenitors of megakaryopoeisis (BFU-MK and CFU-MK).

The constitutive production of TPO occurs primarily in the liver, while the kidneys contribute to a lesser extent (82). Old desialylated platelets bind to the Ashwell–Morell receptor and promote TPO mRNA production (83) (Figure 2). Hepatocytes can also be induced to produce TPO by IL-6 stimulation (84), which likely accounts for the thrombocytosis seen in patients with chronic inflammatory diseases, such as rheumatoid arthritis and ulcerative colitis. However, unlike EPO, TPO levels are for the most part not regulated by changes in gene expression, but rather TPO is taken out of circulation through binding to its receptor and being internalized and degraded (85, 86). Platelets and MKs display the bulk of TPO-R expressed, and so their numbers largely regulate TPO levels (86, 87). While TPO-R expression in mice is dispensable for thrombopoiesis, its expression on MKs and platelets is essential to prevent an excessive production of MKs and other myeloid cells (88). MKs and platelets thus act as a sink *via* the TPO-R, restricting the availability of circulating TPO that could stimulate their production from the pool of progenitor cells.

In thrombocytopenia, there is an insufficient platelet mass to remove TPO from circulation, resulting in high plasma TPO. This feedback mechanism means low platelet numbers result in high levels of TPO, which then stimulates the production of MKs and platelets. Consequently, in the case of *Mpl*^−/−^ mice, where Tpo-R is genetically deleted, high levels of circulating TPO are observed (22). Similarly in humans where TPO-R loss of function causes low platelet levels, high serum TPO accompanies the thrombocytopenia.

This direct regulatory role of platelets, and more specifically TPO-R-binding, on serum TPO levels and production has unexpected consequences: a partial loss of receptor function can counterintuitively result in an increase in platelet numbers. MPL Baltimore (MPL K39N) was the first identified example of this phenomenon, a mutation identified in three thrombocytosis patients. Further investigation found MPL K39N in approximately 7% of African-American populations tested and not at in all populations of European-, Asian-, or Hispanic-American identity (32). Subsequent cases have also been reported in an Afro-Caribbean family in the UK (89), an Ethiopian Jewish boy in Israel (90), and a French woman born in northern Africa (91), suggesting this gene exists in other populations of African origin.

Residue K39 is located in the ECD of TPO-R, and although the structural effect this mutation brings about is not clear, the outcome is reduced protein expression and cell surface localization, observed in both patient platelets and a murine cell line. Another extracellular TPO-R mutation, P106L, is similarly found at a low frequency within a specific population, in this case Arabs (33) and leads to a hereditary thrombocytosis. We and others have sought to determine the mechanisms underlying this latter disease (92, 93).

The Stockklausner study was not able to detect TPO-R P106L on the surface of cells, and therefore put forward a rather unlikely model of TPO internalization not mediated by binding to its receptor on the cell surface, together with TPO-R P106L hypersensitivity to this internalized TPO (92). Nonetheless, we were able to detect TPO-R P106L on the surface of cells using several techniques, and so our conclusions differed from the earlier study (93). Our results indicated that this mutation led to a trafficking defect such that there was low, but detectable, expression of the receptor on the cell surface. This low level of expression is sufficient for signal transduction but not enough for TPO clearance, ultimately resulting in thrombocytosis in patients and in a mouse model.

A similar paradoxical thrombocytosis as a consequence of subnormal receptor expression was previously observed in an attempt to use gene therapy to correct the thrombocytopenia caused by the absence of functional TPO-R at the surface of hematopoietic cells. Lannutti and colleagues unexpectedly found that expressing TPO-R at lower than normal physiological levels in *Mpl*^−/−^ mice led to elevated plasma TPO levels, driving excessive megakaryopoiesis and thrombocytosis (94). A similar finding of thrombocytosis has also been described for *Mpl*^−/−^ mice, where the expression of a *Mpl* transgene was lower than physiological levels in mature MKs and platelets (95). Levels of receptor on the cell surface are therefore a key element in the regulation of TPO-R signaling, and so its traffic and plasma membrane localization must be carefully regulated for normal hematopoiesis.

Mechanistically, paradoxical thrombocytosis can occur when TPO-R is expressed at low levels in late MKs and platelets, due to increased TPO concentrations in plasma that will be able to activate even low levels of TPO-R on early progenitors, such as megakaryoblasts. The basis for this phenomenon is that the amount of activated receptors required for signaling in early progenitors is much smaller than for ligand uptake and degradation (93).

## TPO-R Traffic to the Cell Membrane

It is not fully understood how the trafficking of TPO-R to the cell surface is regulated, although JAK2 and TYK2 are known to promote localization at the cell membrane and stability of the receptor (55). The chaperone effect of JAK2 and TYK2 depends on their N-terminal FERM domains. In complexes with JAK2 or TYK2, the TPO-R cytosolic domain probably masks degradation signals, and the EndoH-resistant mature form accumulates. Interestingly, the cell surface TPO-R pool appears to be even more stable than the intracellular pool (55). TPO-R expression is altered in MPNs, as shown by the downregulation of TPO-R in platelets and megakaryoctes of MPN patients, first described in a classic study from the Spivak laboratory (96). Moreover, TPO-R downmodulation has since been observed to occur in many MPN patients, irrespective of driver mutation (96–99). Further, the cell surface and intracellular levels of TPO-R are decreased by the MPN mutant JAK2 V617F, which inhibits recycling and promotes proteasomal degradation of TPO-R (57).

TPO-R is an N-glycosylated protein and contains four putative N-glycosylation sites in its ECD. The role of N-glycosylation for cytokine receptor traffic and ligand binding does appear to vary across receptors (100–104), but for TPO-R the data suggest that cell surface expression of TPO-R is regulated by the use of a combination of N-glycosylation sites, and that the receptor is able to respond to TPO, once it is localized at the plasma membrane (105).

## A Novel Mechanism of TPO-R Traffic and Activation: Complex Formation with Calreticulin (CALR) Mutants in MPN

The trafficking of TPO-R and how this can be altered in disease has become particularly topical since the discovery that the majority of MPN cases of ET and MF where *MPL* or *JAK2* is not mutated harbor mutations in the gene for the endoplasmic reticulum (ER) chaperone CALR. CALR is a soluble protein that acts as a calcium buffer in the ER, binding calcium ions *via* its negatively charged C-terminal end. CALR also has an essential role in the ER’s protein quality control system as a chaperone, binding to the glycans of nascent N-glycosylated proteins, retaining these glycoproteins within the ER until properly folded (106).

**Figure 3:**
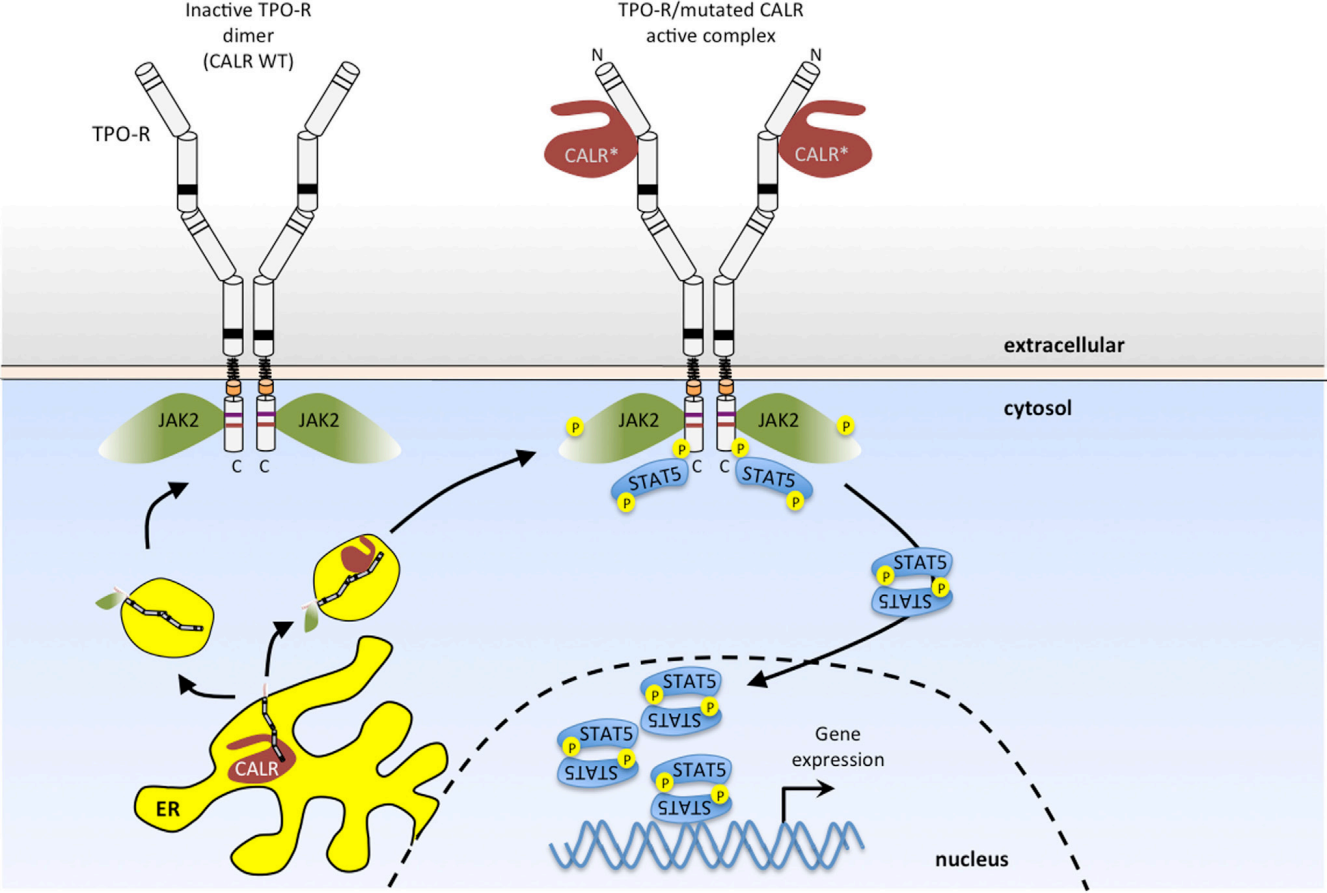
**Myeloproliferative neoplasm (MPN)-associated calreticulin (CALR) mutants bind to TPO-R and activate JAK2 signaling in the absence of thrombopoietin (TPO) ligand**. CALR mutants that result from one base pair shifts in the reading frame in exon 9 have a novel positively charged tail (*). These CALR mutants bind to the N-glycosylated extracellular domain of TPO-R, and such complexes in the secretory pathway and at the cell surface activate JAK2. This results in persistent activation of STAT5 (as shown here) and also STAT1, STAT3, mitogen-activated protein kinase (MAPK)/ERK and phosphatidylinositol-3 kinase (PI3K)/AKT pathways. Wild-type CALR (left), on the other hand, is retained in the endoplasmic reticulum, due to the presence of its KDEL sequence (lacking in MPN-associated mutant CALR) and does not activate TPO-R.

These MPN-associated mutations result in the loss of CALR’s ER retention signal, and the generation of a novel C-terminal sequence due to recurrent +1 frameshifts (107, 108). These mutant CALRs can activate TPO-R in the absence of TPO ligand (98) (Figure 3). That CALR mutants induce JAK-STAT signaling by specifically activating TPO-R has been observed in several systems (98, 109–113), although the details of the specific structural mechanism of TPO-R activation remain to be determined and will require an atomic-level resolution structure of the complex. One possibility is that this mutant CALR fixes the TPO-R dimer in an orientation corresponding to one of the “active” interfaces observed in the coiled-coil fusion experiments described above (51).

Indeed, mutant CALRs induce close proximity (dimerization) of the C-terminal JAK-homology 1 kinase domains of JAK2 *via* TPO-R, as determined by protein complementation assays (98, 109–112). Available evidence indicates that TPO-R activation depends on an interaction with CALR mutants that requires the N-glycosylation sites of the TPO-R ECD and both the lectin-binding domain and the new positively charged tail of CALR mutants (98, 109–112) (Figure 3). Experiments using an array of cytokine receptors (both type I and type II) show that the only cytokine receptor able to support constitutive signaling in the presence of CALR mutants is TPO-R. A weak activation, insufficient to promote long-term proliferation, is also detected with the granulocyte colony-stimulating factor receptor, and no activity with other closely related receptors such as EPO-R, GH-R, prolactin receptor, interleukin, or interferon receptors (98, 109). Expression of CALR mutants in parental Ba/F3 or 32D cells leads to autonomous growth only upon expression of the *Mpl* gene, coding for Tpo-R (98, 109–113). Of note, coexpression with a soluble TPO-R ECD blocked activation of TPO-R by CALR mutants in a dose-dependent manner, providing the receptor was N-glycosylated (98). Detailed analyses further showed that of the four N-linked sugars, the main one mediating interactions with CALR mutants is N117, which is the most distal from the membrane (98).

Coimmunoprecipitation of total and cell surface proteins with CALR mutants demonstrates that they interact more strongly with TPO-R than wild-type CALR (98, 109–112), and that in the presence of CALR mutants, TPO-R is transported to the cell surface in an EndoH-sensitive immature form (98). Confocal immunofluorescence and cellular fractionation show that CALR mutants exit the ER and are located in a distinct pattern from that of wild-type CALR, mainly in the ER-to-Golgi vesicles and at the cell surface (98). In addition, CALR mutants appear to be much more unstable than the wild-type CALR (98, 109–112).

The consequence of persistent activation of TPO-R is activation of JAK2/STAT5/STAT3/STAT1 and also of the other JAK2-dependent pathways, MAP-kinase/ERK and PI3K/AKT. While JAK2 and PI3K inhibitors synergize to inhibit proliferation of JAK2 V617F cells, in the case of CALR mutant transformed cells, JAK2 inhibitors synergized with ERK inhibitors, suggesting subtle differences in the levels of activated downstream pathways (98, 109–112). *In vivo*, expression of CALR mutants leads to pathologic MK proliferation, resulting in an ET phenotype, which can progress to MF (98, 109–112). While all pathogenic CALR mutants possess the +1 frameshift, small differences in the preceding sequence to the novel positively charged tail exist, and the class of mutants represented by del52, where less of the initial negative charges remain on the final protein, appear to signal more strongly and lead to a more pronounced myeloproliferation phenotype (98, 109–112).

The results obtained in cell lines transduced with retroviral vectors expressing CALR mutants were recently confirmed by creating endogenous heterozygous and homozygous CALR mutants using the CRISPR/Cas9 system (96–99, 109–112). Interestingly, in such cells it is observed that the cell surface levels of TPO-R are downmodulated and that while there is constitutive activity of TPO-R, response to TPO is slightly decreased, consistent with such downregulation (96–99).

Overall, this is a novel and fascinating mechanism of oncogenesis whereby a mutated chaperone transforms cells by persistently activating a specific cytokine receptor, namely TPO-R (Figure 3), fitting well with the involvement of CALR mutants in pathologic MK proliferation.

## Receptor Agonists

Since the discovery of TPO as the ligand for MPL, there has been significant clinical interest in its use as a stimulator of platelet production. First-generation forms of TPO used in clinical trials were developed almost 20 years ago, primarily a recombinant human TPO and a PEGylated TPO-derived peptide (PEG-rHuMGDF). Both of these treatments showed very positive effects (114, 115), increasing thrombopoiesis in both thrombocytopenic and healthy subjects. Despite these initially promising results, neither drug trial continued as some healthy subjects treated with PEG-rHuMGDF-developed thrombocytopenia due to the production of antibodies that cross-reacted with endogenous TPO (116, 117).

The development of second-generation thrombopoietic agents has accordingly aimed at minimizing structural similarities with TPO. These TPO-R agonists can be classified into three types: TPO peptide mimetics, TPO non-peptide mimetics, and TPO-R antibodies.

Two receptor agonists are currently licensed by the FDA and the EMA: romiplostim (AMG 531; Nplate, Amgen) and eltrombopag (SB-497115; Revolade, GlaxoSmithKline). Romiplostim, a TPO peptide mimetic, was developed from a peptide library screen that identified a 14-amino acid peptide with no homology to TPO, yet high affinity for TPO-R and capable of stimulating a TPO-responsive cell line (118). Eltrombopag, on the other hand, does not bind the receptor in the same manner as TPO, but rather targets TPO-R’s TMD, or more specifically its extracellular juxtamembrane end. Its mechanism of action appears to derive from inducing helical secondary structure around the TMD residue H499, where it binds altering the positioning of the receptor dimer to an activating conformation that exhibits H499 stabilized outside the dimer interface (48).

Romiplostim and eltrombopag are licensed for the treatment of chronic immune thrombocytopenia and thrombocytopenias of liver diseases, such as hepatitis C infection, but another potential target for treatment with receptor agonists is CAMT. There are many different mutations in TPO-R that lead to CAMT, and apparently a number of ways for TPO-R function to fail. Several studies have sought to differentiate the various types of failure in the hope that this could define specific therapeutic strategies for individual patients, depending on the mutation they bear.

While nonsense and frameshift mutations can be assumed to produce truncated receptor, lacking essential domains for signal transduction, it is more difficult to predict the effect of missense mutations. We and others have sought to determine what sort of function is altered by each particular mutation in TPO-R (41, 42, 119). Most of these missense mutations identified in CAMT patients are found in the ECD of TPO-R, and from these studies, we can glean that for the most part these mutant receptors fail to signal due to lack of presentation on the cell surface (42). There appear to be several different defects that contribute to this outcome, including a failure to express at an mRNA level, reduced expression at a protein level, and improper N-glycosylation (119). Some CAMT TPO-R mutant proteins do nonetheless make it to the cell surface, and cells expressing these mutant receptors either do not respond or have a severely perturbed response to TPO stimulation (41, 42). A pertinent question is, then, are these receptors able to signal when treated with receptor agonists? And could CAMT patients with these particular mutations benefit from this type of therapy?

One study examined the effect of receptor-targeting agonists murine Amgen Megakaryopoiesis Protein 4 (m-AMP4; Amgen), a version of romiplostim effective on murine TPO-R, and LGD-4665 (Ligand Pharmaceuticals), a small molecule non-peptide TPO-R agonist, effective on CAMT TPO-R mutant receptors (41). They found that cells expressing the CAMT mutant TPO-R F104S did not proliferate in the presence of TPO or the ECD targeting m-AMP4 but did respond to the transmembrane targeting LGD-4665 small molecule. The results of this study demonstrate that these receptor agonists are not interchangeable, and knowledge of the mutation driving the disease could inform treatment choice. Despite promising early results, this latter drug nonetheless does not seem to have progressed to a phase III trial.

The third group of TPO-R agonists with potential in the clinic is receptor antibodies, including minibodies and diabodies. Minibodies are single-chain antibody fragment dimers, while diabodies are non-covalent dimers of two covalently linked antibody fragments. These alternative approaches involve identifying antibodies that activate receptor function. Orita and colleagues (120) produced two novel TPO-R agonists, one that had activity in a megakaryocytic cell line, as well as *in vivo* in cynomolgus monkeys, and a second which increased proliferation in TPO-dependent cell lines expressing TPO-R with mutations found in CAMT patients. There is also interesting evidence from work on diabodies targeting the EPO-R ECD that these receptor agonists are able to induce different signaling responses, as a function of the distances of separation imposed on receptor monomers (121), suggesting this might also apply to TPO-R, given that several active dimer interfaces have been identified for the latter receptor (51).

## Receptor Antagonists

Efforts to dampen, rather than activate, TPO signaling with antagonists of TPO-R have only recently been described (80). LCP4, a 20-amino acid cyclic peptide TPO-R antagonist, was used to treat CD34+ cells derived from splenic, peripheral blood, and bone marrow cells of MF patients. Although TPO promoted the proliferation and differentiation of these cells, LCP4 was able to inhibit these effects, more profoundly in cells from MF patients than healthy controls, effectively depleting MF HSCs and progenitor cells (80). This new class of drug has exciting potential to target HSCs bearing activating mutations, while sparing normal HSCs.

## TPO-R and EPO-R: Some Comparisons

Belonging to the same homodimeric type I subfamily of cytokine receptors and activating the same JAK2 tyrosine kinase, TPO-R and EPO-R nonetheless exhibit significant differences in their induction, expression pattern, biology, mechanism of activation, and signaling. TPO-R is expressed on HSCs, early myeloid progenitors, and MK progenitors, as well as on the eventual differentiated platelets, while EPO-R is expressed on erythroid progenitors (but not beyond basophilic erythroblasts and not on mature red cells), on endothelial cells, and on neurons. TPO-R regulates platelet numbers that can increase by twofold to fivefold in certain pathological conditions, while EPO-R regulates the hematocrit, which has a much smaller range of increase (above the normal values of 42–44%). EPO-R is predominantly a preformed dimer, at least in transfected or transduced cells (122, 123), and its transmembrane sequence forms dimers with great efficiency (122, 124, 125), that is significantly higher than that of TPO-R TMDs (50). For the latter, preformed dimers have also been detected (45, 50), but overall the extent of predimerization appears lower and a monomer-dimer equilibrium might be operative in cells expressing physiological levels of TPO-R.

Activation of TPO-R has been reported by many mechanisms, including mutations in transmembrane and juxtamembrane domains, leading to disease-causing mutations (126). Also, artificially one can activate TPO-R quite easily, with antitag antibodies (127) or by small molecules (for example, eltrombopag). In engineered constructs, it appears that several dimeric interfaces imposed on the TMD can lead to TPO-R activation (50, 51). This is not the case for EPO-R, which requires one specific interface for activation (36), and in which no activating transmembrane or juxtamembrane mutations have been reported in blood diseases. An exception is observed in acute lymphoblastic leukemia, where translocations of EPO-R with high expression levels have been recently described (128). Finally, an additional point of difference that has come to light is that mutated forms of CALR have been shown to specifically activate TPO-R but not EPO-R (98).

## Conclusion

Since its discovery over a quarter of a century ago, our understanding of how TPO-R functions has gradually improved. While attempts to develop a detailed structural model of the receptor based on direct experimental evidence has had limited success, it has been possible to exploit knowledge of related receptors for homology-based predictions, as well as utilizing indirect mutagenesis studies. Despite this lack of structural information, the receptor has been extensively characterized and two small molecule receptor-binding agonists have been developed and approved for use in the clinic. The production of diabodies to modulate TPO-R activity is a promising potential addition to the currently available treatments. We are hopeful that the coming few years will bring further insight into TPO signaling, particularly from a structural viewpoint, which could inform more targeted approaches in the design of therapeutics to treat thrombocytosis and other hematological disorders driven by abnormal TPO signaling.

## Author Contributions

LV wrote review and edited review and figures. J-PD and CP edited review and produced figures. SC wrote sections of review and edited review and figures.

## Conflict of Interest Statement

The authors declare that the research was conducted in the absence of any commercial or financial relationships that could be construed as a potential conflict of interest.
